# Integrative research and innovation strategy for rare diseases. Insights from the 5-year European joint programme on rare diseases, including analysis to inform recommendations for future actions

**DOI:** 10.1186/s12961-025-01389-7

**Published:** 2025-10-16

**Authors:** María del Carmen Sánchez-González, Rodrigo Sarmiento-Suárez, Laura Lee Cellai, Clément Moreau, Marcin Bartoszewicz, Piotr Fiedor, Domenica Taruscio, Marta de Santis, Manuel Posada de la Paz, Daria Julkowska, Claudio Carta, Eva Bermejo-Sánchez

**Affiliations:** 1 Institute for Rare Diseases Research (IIER), Instituto de Salud Carlos III (ISCIII), Madrid, Spain; 2 Istituto Superiore Di Sanità, Rome, Italy; 3 Inserm, Paris, France; 4 Medical University of Warsaw, Warsaw, Poland; 5 University of Economics and Human Sciences in Warsaw, Warsaw, Poland

**Keywords:** Rare diseases, National plans, Innovation strategy, Research ecosystem

## Abstract

**Background:**

The European Joint Programme on Rare Diseases (EJP RD) was an initiative that sought to integrate different rare disease strategies into a research ecosystem. This paper summarizes the work of the dedicated work package on Integrative Research and Innovation Strategy and outlines recommendations to facilitate alignment with National Plans and Strategies for Rare Diseases.

**Methods:**

We carried out three periodic surveys (in 2020, 2021 and 2023) on the status of national plans and strategies (NP/NS) for rare diseases in EJP RD member countries. Using the feedback from the surveys and other sources, we developed an annual mapping of research and innovation needs and their alignment with the EJP RD programme. A review of the findings and progress from these approaches – including two strategic policy workshops and the development of National Mirror Groups – informed the recommendations to bridge the gap between national efforts and European strategies.

**Results:**

A total of 34 countries responded to at least one survey. Implementation is uneven: 76% of countries have had their NP/NS approved at some point, but renewals are irregular. For EU13 countries, the most frequent barrier to developing, improving and translating rare disease (RD) research results was funding. In terms of the Programme’s activities covering mapped needs, we achieved global coverage of 65.8%, with greater coverage of needs for both the diagnostic pathway and the treatment pathway (71%). Four National Mirror Groups were developed during the Programme’s first 5 years, and a further seven were established by mid-2024.

**Conclusions:**

Despite recent progress in establishing a research ecosystem for rare diseases in Europe, several challenges remain and should be addressed. These include availability and accessibility of diagnostics, medicines and medical devices across Europe, inequalities between and within countries, compliance with the FAIR data principles and the lack of a comprehensive policy framework to integrate different rare diseases initiatives. Next actions need to strengthen the coordination and alignment of funding and national policies, innovation in the translation of research results and the reach of a holistic research ecosystem. National Mirror Groups will play an important role in this respect.

**Supplementary Information:**

The online version contains supplementary material available at 10.1186/s12961-025-01389-7.

## Background

Rare diseases (RD) are a variety of diseases whose prevalence is less than 5 per 10 000 people as has been defined by the European Union (EU). Despite being rare, such diseases affect nearly 6–8% of the European population, as more than 6000 different conditions have been described according to the Orphanet portal [1]. Most people affected by RD face long diagnostic journeys, as the rarity of the event leads the patients to many loopholes in the diagnostic process [2].

The European Joint Programme of Rare Diseases (EJP RD) [3] is an EU initiative that seeks to integrate different RD projects and programs into a research ecosystem aimed at improving the translation of results to accelerate the diagnosis and treatment of patients with RD, which is the most pressing issue for the patients and their families.

EJP RD objectives are intertwined with the three goals for the decade 2017–2027 of the International Rare Diseases Research Consortium (IRDiRC) [4], which is one of the leading strategies to boost rare disease research and knowledge translation. Goals are the following: (i) to reach a diagnosis for all patients with rare diseases within the first year of coming into medical attention, (ii) the development and approval of 1000 new therapies and (iii) the design and development of methodologies to assess the impact of diagnoses and therapies for patients with rare diseases. The EJP RD work package (WP 2) “Integrative Research and Innovation Strategy” has developed a strategy to foster the achievement of these goals, which include the following activities: (i) mapping research and innovation needs, (ii) defining the prioritization model for EJP RD actions, connecting them with sustainability and ethical, regulatory and legal frameworks, (iii) prioritizing topics for Joint transnational calls (JTC), (iv) feeding medium- and long-term research and innovation strategy in collaboration with IRDiRC and (v) translating the impact on national and EU RD plans and strategies.

This paper summarizes and presents the work carried out by the EJP RD WP2 on “integrative research and innovation strategy” from 2019 to 2023. It describes the activities developed to identify needs and analyses the progress made, as well as provides recommendations for actions to improve alignment between national strategies and the evolving RD research ecosystem, including policies and other nonscientific domains. Particular attention is given to the establishment of National Mirror Groups (NMGs) by EU countries to promote adequate research and healthcare for the RD community from a holistic perspective.

## Methods

The work carried out during the EJP RD has followed three distinct approaches, as defined below.

A mapping of research and innovation (R&I) needs was carried out annually^1^ by gathering information from the RD community and literature, input from the governing bodies (including policy boards) of the EJP RD and feedback from surveys of EU Member States (MS) and other EJP RD countries. The processes for identifying the relevant topics for the annual JTC were also integrated. The needs identified over the years were classified according to their pathway (diagnosis, treatment, both and other related nonscientific domains, including diagnosis and healthcare, regulatory and ethics and EU competitiveness and innovation) and in relation to the vision and goals of IRDiRC.

The information from the RD community and literature included several sources, such as EURORDIS’ information [5], in the form of actions and barometer consultations, (patients’ perspective); IRDiRC’s international research strategy; data from the R&I Days launched every year by the Directorate General for R&I of the European Commission & Horizon Europe [6], European infrastructures^2^ and initiatives such as the Joint Action Towards the European Health Data Space Joint Action (TEHDAS JA) [7], European Open Science Cloud (EOSC) [8], Innovative Medicines Initiative (IMI) [9] projects and Horizon Europe partnerships candidatures [10]. In addition, policies such as the Orphan drug regulation’s update [11] and statements such as the United Nations’ Resolution on RD [12] fed the mapping.

**Fig. 1:**
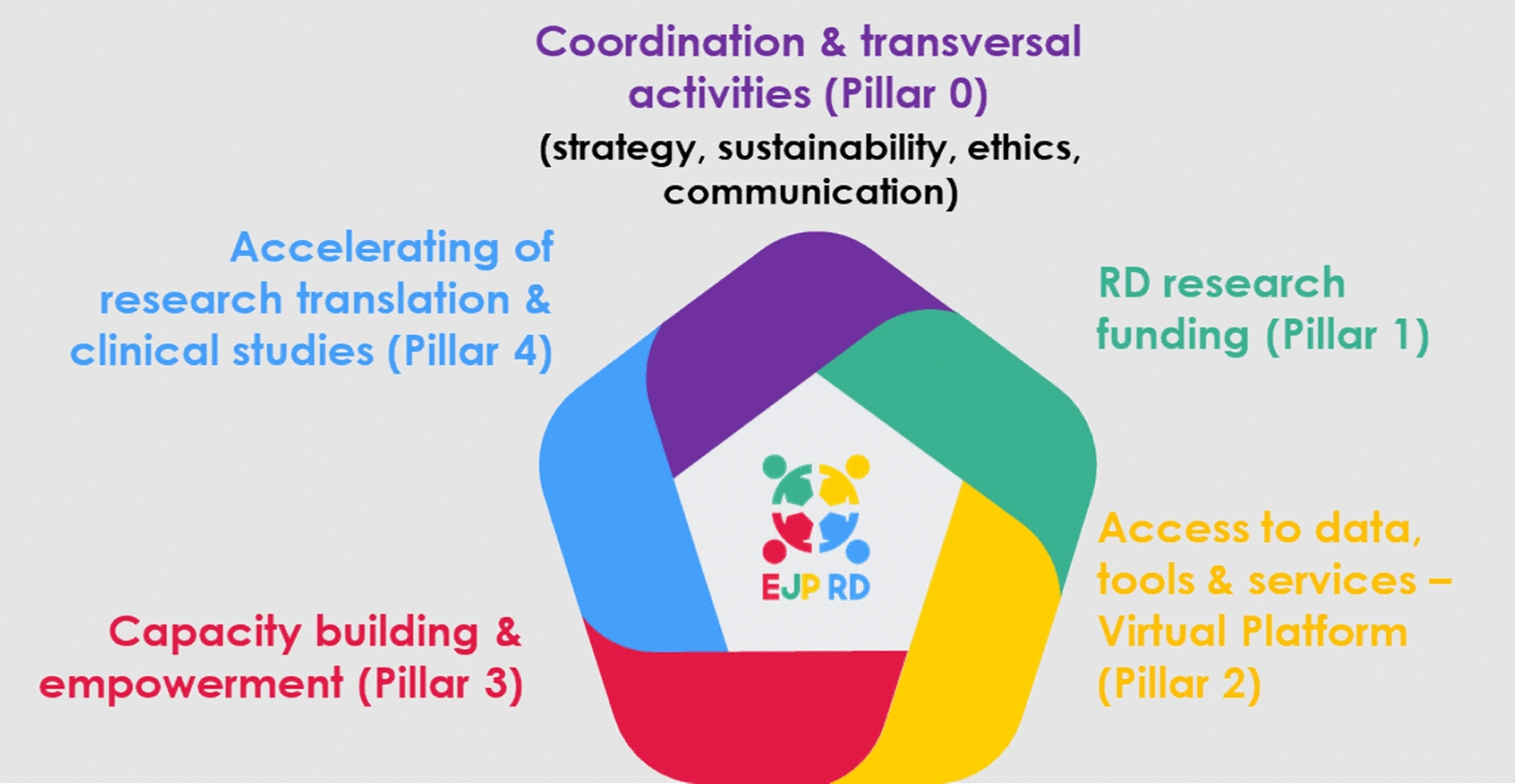
Pillars of the EJP RD: Source: dissemination material from EJP RD

A periodical collection of information from EU Member States and other EJP RD members via a survey,^3^ targeting National Mirror Groups or persons directly involved in national RD actions, on the EJP RD relevant/complementary actions performed at national level, was also carried out. Particular focus was set on EU13^4^ countries in respect to their specific needs, obstacles and advancements. The survey analysed the status quo regarding the existence of National Plans and Strategies (NP/NS) for RD as key instruments to reach a common RD strategy at national and European level. Moreover, the survey analysed the matching of the initiatives proposed by the NP/NS for RD, and of other national/international RD actions of each country with the activities of the four major EJP RD pillars (Fig. 1). All the surveys of this series of data collection were composed of multiple-choice questions and open questions, divided into general and specific sections dedicated to NP/NS for RD and other relevant initiatives, the alignment with EJP RD and a focus on EU13 countries. Participants only had to fill in updates in subsequent editions. The original questionnaire is available in Additional file 1 “Original Survey”.

The three editions of the survey on NP/NS for RD have been spread in years 2, 3 and 5 of the EJP RD (years 2020, 2021 and 2023), reaching a total of 36 countries (Armenia, Austria, Belgium, Bulgaria, Canada, Cyprus, Croatia, Czechia, Denmark, Estonia, Finland, France, Georgia, Germany, Greece, Hungary, Ireland, Israel, Italy, Latvia, Lithuania, Luxembourg, Malta, Norway, Poland, Portugal, Romania, Serbia, Slovakia, Slovenia, Sweden, Spain, Switzerland, the Netherlands, Turkey, and the United Kingdom).

Ideally, the target of the survey was the NMG,^5^ as they are key actors in identifying, discussing and bringing national needs to the attention of those at a decision-making level. Where there was no NMG, members who were deeply and directly involved in national RD policies were contacted instead.

The answers submitted by the countries that participated in the survey in 2023 and in at least one of the editions 2020/2021 were also compared to draw a follow-up of the evolution of the alignment status of these countries with the EJP RD activities over the years.

The data gathered through the surveys directed at the EU MS and other EJP RD countries were presented in periodic reports (public deliverables) analysing the national alignment with EJP RD and proposed at the occasion of the Policy Board & ExCom meetings. These outcomes, monitoring the impact/translation of the prioritization on national and EU strategies, have been considered to shape the Annual Work Plans to provide for informed and context-aware inputs for the future EJP RD actions. Moreover, the results have been presented in the context of two Strategic Workshops^6^ with relevant policy stakeholders, having the aim to feed coordinated undertakings at national, European and international levels to create a virtuous RD research ecosystem. This input also informed the mapping of R&I needs. The continuous review and analysis of the findings and progress from these approaches informed the recommendations to bridge the gap between national efforts and European strategies.

## Results

The results of the surveys carried out over the years and their relationship with the R&I needs identified during the programme point the way towards an integrated research and innovation strategy. These results, presented in a series of public deliverables [13–16], are aggregated according to the EJP RD pillars “RD research funding” (pillar 1), “access to data, tools and services-virtual platform” (pillar 2), “capacity-building and empowerment” (pillar 3) and “accelerating of research translation and clinical studies” (pillar 4), as well as follow-up (trends over the years). Results for the 2020 and 2021 surveys are presented in a table at the end of the section (Table 2).

The specific features of EU13 countries are mentioned separately in each section. Information on the workshops, the activities carried out by EJP RD to address the needs and details on NMGs, conclude the presentation of the results.

### Surveys and Research and Innovations Needs

In all, 34 of the 36 contacted countries^7^ participated in at least one edition of the survey. Of the 34 responding countries, 13 (36%) participated in one edition, 8 (22%) in two editions and 13 in three editions (36%). When considering the EU13 countries, 100% were represented, with all EU13 countries participating in at least one edition of the survey. Three countries participated in all editions (23%), four countries in two editions (31%) and six countries in at least one edition (46%).

#### General information, overall alignment status and status quo of NP/NS for RD

The first edition, in which 21 countries participated, enabled an early status quo both on the existence and state of NP/NS for RD and on the alignment of these plans and strategies (as well as of other national RD undertakings) with the activities promoted by the EJP RD. This partial participation has been related to some extent to the burden faced by health institutions for the *force majeure* situation of the coronavirus disease 2019 (COVID-19) pandemic. The results showed, nevertheless, a general heterogeneity in the state of advancement/implementation of national polices, plans and strategies for RD (existence, date of approval, expiry, periodical evaluation), and pointed out areas of strengths, needs and gaps in the national activities when referring to the actions promoted by the four EJP RD pillars.

Besides the general adoption at some stage of a NP/NS for RD by the participating countries (76%), in 37.5% of these countries the NP/NS for RD were expired and seemed not in the process of renewal/not clear if under renewal.

To the second edition of the survey, spread over 2021, and 6 months later than the first edition, six of the countries that did not reply to the first survey joined the data collection.^8^ In this way, a total of 27 countries was considered in this second edition, summing up the new responding countries and the countries that confirmed/updated the previously given information.

The third edition of the survey, distributed in 2023, was analysed with the dual objective of collecting the state of the art at year 5 of the EJP RD, and of drawing a follow-up analysis to describe the evolution of the matching faced by the countries participating in the EJP RD with the projects’ activities over the years.

All the countries whose NP/NS for RD were time bound had expired and were not replaced by a new edition. No information on the eventual development of a following edition was gathered by the survey.

A total of 25 countries^9^ participated in the last data collection. The results obtained considered all 25 countries participating in the third edition and were in line with the global trend observed in previous data collections, although some differences could be spotted when analysing the results of the alignment status with the 4 EJP RD pillars in more detail.

**Fig. 2:**
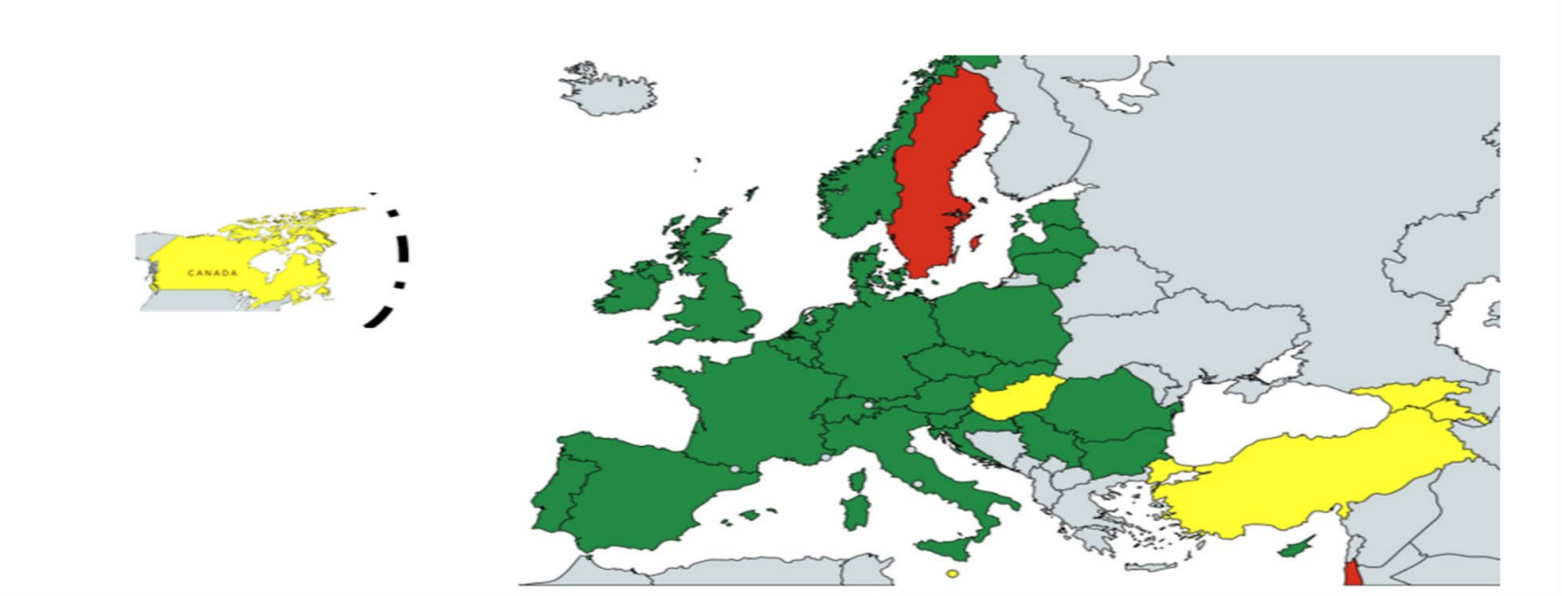
Period 2020–2023: state of the NP/NS for RD according to the survey (34 countries). Green countries with active NP/NS, yellow countries with NP/NS expired and/or under development, red countries without NP/NS; source: internal dissemination materials from EJP RD

In total, 96% of the countries demonstrated that they had NP/NS for RD that at the time of the data collection was either active, expired, under renewal, approval or in development. Only 44% of these NP/NS for RD were approved and in force; 4% of the countries had NP/NS for RD approved but not in force; 12% of the NP/NS for RD were expired and not under renewal, whilst 20% were expired and under renewal; 8% of the countries had developed NP/NS for RD that were in the process of approval; and the remaining 8% of the countries declared that the NP/NS for RD was under development. In addition, 4% of the countries declared to not having provided NP/NS for RD (neither active, nor expired, under approval or development). Figure 2 collates the status for the period 2020–2023.

Focussing on the EU13 countries, seven participated in the 2020 survey (Bulgaria, Czechia, Estonia, Hungary, Lithuania, Romania, Slovakia). In 2021, Croatia, Latvia and Poland added their responses. Regarding the situation of NP/NS, two EU13 countries (20%) were in the process of developing NP/NS for RD, whilst the other EU13 countries (80%) appeared to have expired NP/NS for RD. The 2023 data collection, with 10 countries,^10^ showed that 30% had an active NP/NS for RD, 50% had an expired NP/NS for RD, 10% had developed a NP/NS for RD that at the time of the data collection was in the process of approval and 10% had a NP/NS for RD that had been approved but was not in force.

Overall, identifying and addressing the specific needs of the EU13 is a necessity in itself. In this sense, the need to balance the performance of health and research systems and reduce the gap on R&I of the widening countries (EU13) was recognized at the European Research and Innovation Days in 2022 [6]. In addition, the lack of a critical mass and role of patients with RD affects the widening countries especially. Finally, other transversal needs for EU13 countries worth mentioning are language barriers and absence of options for exploitation at the national level that can affect the interaction between countries and therefore the implementation of NP/NS.

#### Alignment of the countries’ activities per pillar over the years

##### RD research funding (pillar 1)

In the 2023 edition, 58% of the NP/NS for RD appeared to promote national calls for research projects, 46% promoted joint transnational calls for research projects and 38% promoted investments to share knowledge. Table 1 presents JTC topics for all years. Other public funding initiatives for research and/or networking have been highlighted in 16% of the countries, and private funding initiatives in 12%.

**Table 1:** Joint transnational topics

Launch year	JTC Topic^a^
2019	Acceleration of diagnosis (e.g. for undiagnosed patients, variants and development bioinformatic tools) and/or explore disease progression and mechanisms of rare diseases (e.g. natural history studies)
2020	Pre-clinical research to develop effective therapies for rare diseases, that is, studies on preclinical settings, with small molecules, repurposing, disease models, biomarkers or proof-of-concept studies
2021	Social sciences and humanity research to improve healthcare implementation and everyday life of people living with a rare disease. Interdisciplinary collaboration
2022	Development of new analytic tools and pathways to accelerate and facilitate diagnostic monitoring of rare diseases. Interdisciplinary collaboration
2023	Natural history studies, to collect and analyse comprehensive patient data to define targets for future therapies, taking into consideration innovation, safety and efficacy

*JTC* Joint Transnational Call; ^a^All yearly JTC calls (2019–2023) were prepared with collaboration of funders, participation of experts and engagement of the patients on the basis of the perceived needs identified by the RD research community

The promotion of both national and transnational calls for research projects by the NP/NS for RD has been shown by 30% of the EU13 countries, and in 10% towards investments to share knowledge.

Regarding national initiatives other than the NP/NS for RD, in 30% of the EU13 countries there were other public funding initiatives that promoted national calls for research projects, in 10% private funding initiatives and in 20% investments to share knowledge. Funding, including knowledge of available sources, is one of the most crucial needs for EU13 countries [15, 17]. The need for “long-term sustainable investment in the European Research Infrastructure ecosystem to contribute to excellence in basic research, with an increasing investment” identified by the EU Council can be applied to the RD ecosystem [18].

In addition to the commitment of public funding, sustainability also requires private capital investment, strong involvement of industry (large and SMEs) and the reduction of the innovation gap between countries to enable local tailor-made innovation policies.

There is a requirement for resources and sharing optimization as well (multipurpose registries, cluster registries, epidemiological platform and infrastructures).

##### Access to data, tools and services-virtual platform (pillar 2)

The mapping of research and innovation needs in 2020 and 2021 revealed the necessity of specific RD policies, as shown by the 2030 project’s knowledge base. These policies are one of the main drivers of RD strategic policy framework alongside the recognition of RD challenges and the inclusion of patients in the policymaking process, amongst other factors [19].

Several needs on data and tools were mapped during this time, including those mapped from the pillar 2 survey of researchers from European Reference Networks (ERNs) [20]. Researchers found that the ability to locate biobanks/biosamples/cell lines, support for translational research and adequate registries were essential needs. The differences between ERNs and researchers, likely due to different type of research, were found in the most relevant services for them: whereas ERNs declared support to clinical trials and registries as the services of utmost relevance for them (doubling researchers’ answers), the facility to find animal models seemed more relevant for researchers than for ERNs, when compared. Needs to tackle barriers such as complex procedures and legal issues (i.e. legal jurisdiction), and use of data were also extracted from these results.

FAIRness (from Findable, Accessible, Interoperable and Reusable-FAIR principles) is a recognized need for data. In fact, one of the knowledge bases uncovered by the 2030 project [19] is “data collection and utilization” and one of the potential drivers is the FAIRness, covering the horizon scanning trend 9, or need for increased potential for large sets of standardized and interoperable data. Regarding data sharing, specifically in ERNs, there was an identified need for strategic and operational considerations, including the prioritization of data-sharing scenarios, with technological and infrastructure requirements to support them [21].

On the line of the mapping of activities of resources and services to foster research, which was described above for the first round of surveys, parallel to the second round of the survey in 2021, the European Rare Disease Research Coordination and Support Action Consortium (ERICA) was set up to enhance rare disease networks as the ERN and therefore improve data collection strategies and develop high-quality clinical trials with closer involvement of patient organizations [22]. In addition, the Board of Member States on ERN highlighted the need for standardization and FAIRification of registries and platforms, as there is high heterogeneity across the different ERN [23]. Further, IRDiRC has developed several specific task forces to tackle the gaps in diagnosis and treatment include the development of specific task forces, such as: (i) Telehealth for RD, whose purpose was to identify barriers and opportunities for its use in RD; (ii) the Drug Repurposing Guidebook, aimed at providing guidance and tools on drug repurposing; or (iii) the PLUTO project (Disregarded Rare Diseases) that addresses the scarcity of research for extremely rare diseases [24, 25]. Similar to the other pillars, the results of the 2020 and 2021 surveys are presented in Table 2.

**Table 2:** Results of the 2020 and 2021 editions of the survey, by pillar

Survey year	2020*		2021**	
Pillars	EJP RD^£^	EU13^¥^	EJP RD^£^	EU13^¥^
RD research funding (P1)				
National calls	44%	UKN	59%	33%
Joint transnational calls	56%	17%	46%	33%
Investments to share knowledge	44%	33%	46%	33%
Other public funding/networking	81%	67%	68%	56%
Private funding initiatives	56%	33%	48%	11%
Access to data, tools and services (P2)				
Advisory body of national experts				
General	50%	50%	52%	56%
Specific for RD	13%	1%	8%	1%
Support data repositories and tools for RD in NP/NS				
General support	81%	50%	78%	56%
FAIR data	31%	NR	50%	NR
Holistic approaches RD diagnosis and treatment	72%	50%	81%	67%
Initiatives other than NP/NS				
Public investment	50%	17%	44%	22%
Private funding	38%	17%	48%	12%
FAIR data	25%	17%	44%	11%
Holistic approaches RD diagnosis and treatment	NR	33%	44%	45%
Capacity-building and empowerment (P3)				
Promotion/support of RD trainings by NP/NS				
All trainings	75%	67%	78%	67%
Empowerment of patients	83%	67%	72%	56%
Registries	42%	50%	61%	56%
Online education courses	42%	50%	39%	44%
Data management	33%	33%	44%	33%
Standards and quality of data (clinical and lab)	33%	67%	39%	56%
Biobanks	22%	17%	28%	22%
Data quality	22%	33%	22%	22%
FAIR data	NR	NR	11%	NR
Initiatives other than NP/NS (All trainings)	75%	NR	56%	NR
Accelerating research translation (P4)				
Rapid translation of research results	50%	17%	48%	33%
Innovative methodologies for CT	56%	17%	61%	11%
Other initiatives than the NP/NS	38%	17%	36%	NR

*CT* clinical trial, *NP* national plan, *NR* not reported, *NS* national strategy, *UKN* unknown (four countries had negative answer and two “I do not know”) *21 countries (7 EU13); **27 countries (10 EU13); ^£^ EJP RD partners including EU and associated partners; ^¥^ Only EU13 countries

From the third data collection (2023), the alignment status of the countries with the activities of pillar 2 unfolded as follows: 72% of the countries informed as being provided with a non-RD specific advisory body of national experts for research and innovation, and 7% with such an advisory body, specific for RD; 63% of the countries referred that their NP/NS for RD supported data repositories and tools for research on RD, 36% that it supported FAIR data, and 84% the adoption of multidisciplinary holistic approaches. Other public funding initiatives for the support to data repositories and tools for research on RD were found in 24% of the countries, and private funding initiatives in 20%. For the support to FAIR data, 24% of the countries had other initiatives that promoted them, whereas the fostering of other initiatives to the adoption of multidisciplinary holistic approaches for RD diagnostics and therapeutics has been highlighted in 24% of the countries. Regarding R&I needs for this period, ERICA continued their work on strengthening ERNs and carried out a workshop on ERN registries and data management strategies highlighting the importance of data use in RD. At the same time, data sharing and re-use was at the centre of the discussions of the Innovative Medicines Initiative (IMI) [26].

Considering the EU13 outcomes of the survey in 2023, 40% of the countries had an advisory body of national experts for Research and Innovation and 10% had an advisory body specific for RD. In addition, 80% of the EU13 countries had NP/NS for RD that supported data repositories and tools for research on RD, 80% the adoption of multidisciplinary holistic approaches for RD, and 50% FAIR data. Further, 10% declared the presence of other public funding initiatives than the NP/NS for RD that fostered data repositories and tools for research on RD, and the same percentage of private funding initiatives.

On the contrary, 20% of the EU13 countries showed other initiatives for the support to FAIR data, and 30% when considering other initiatives for the adoption of multidisciplinary holistic approaches for RD diagnostics and therapeutics.

The holistic approach has emerged as a continuous need over the years, not only for the EU13 countries [27]. As reflected in the mapped needs of the nonscientific domains of diagnosis and healthcare, the gap between medical innovation and community healthcare should come to an end [28, 29]. This holistic approach, based on the needs of the person and on better access to high-quality medical and social care, is an essential need [30]. At the same time, another need that remains important is the encouragement of FAIRness in data management.

##### Capacity-building and empowerment (pillar 3)

The need for training had been identified by EURORDIS in previous years [31], particularly in natural history studies and underlying mechanisms, for researchers at all stages of their career. In addition, training is needed for developers of therapies in the drug development pathway, especially in regulatory aspects.

In the first survey (2020), no countries indicated support for training on FAIR data through the NP/NS for RD. Additionally, the results of the pillar 2 survey (2019) of ERNs [20] revealed a lack of general understanding of standards and data FAIRification concepts, and therefore a need for FAIRness training was identified.

In addition, the mapped needs for training in that period showed that education is a tool to connect innovation, research and business [6]. The needs for capacity-building included the training for trainers, a necessity to adapt to national schemes. This pillar is a channel for training and the coaching of more involved or experienced institutions and countries. The inclusion of early career researchers in calls or networking schemes is also valuable and thus a mapped need. Table 2 presents the results of the surveys conducted during this period.

In the third edition of the survey (2023), 84% of countries responded that they promote RD training through the NP/NS for RD. The areas covered by the trainings were, in descending order of frequency, “empowerment of the patients” (70%), “registries” and “online education courses” (both at 45%), “standards and quality of genetics/genomics data in clinical practice and laboratories” (35%), “data management” and “data quality” (both at 25%), “biobanks” (15%) and “FAIR data” (10%). In 24% of the countries the presence of other activities to support RD trainings was stated.

It is worth noting that the need for specific training on socio-health aspects, especially on the resources available in the health system for this care pathway, was mapped as well. Regarding the needs in capacity-building, the EJP RD ERN research fellowships, which started as an initiative from the ERN Board of Member States, facilitate the mobility of trainees along different ERN and therefore attract young researchers to the RD field. The empowerment of patient organizations, which has grown in the last years, is also worth mentioning, as it has urged the European Commission and the Member States, through the campaign Rare 2030 Action base on the recommendations of the Rare 2030 Foresight Study, to adopt an action plan for RD that should include: (i) avoiding delays in diagnosis, (ii) reducing premature deaths due to RD, (iii) reducing economic, social and psychological burden of RD and (iv) fostering European-led R&I [32, 33].

In addition, there is increasing participation of patients in RD research, and studies in this area have been more oriented to patients’ needs [17].

For the EU13 countries, all the responses to the 2023 edition revealed endorsement of training activities through the NP/NS for RD. The training activities backed by the NP/NS for RD comprised (in descending order of frequency): “empowerment of the patients” (90%), “registries” (60%), “online education courses” (50%), “data management” (30%), “data quality” (30%) and “standards and quality of genetics/genomics data in clinical practice and laboratories” (30%), “FAIR data” and “biobanks” (10%).

Finally, 20% of the EU13 countries stated that they promote training through initiatives other the NP/NS for RD.

Amongst the barriers faced by EU13 countries during the first and second surveys, the imbalance between initiatives of capacity-building and countries’ R&I needs was highlighted. Furthermore, there is a need to increase networking strategies to overcome the fragmentation of activities in the field of RD in these countries [17].

##### Accelerating of research translation and clinical studies (pillar 4)

The needs related to this area were the main objectives of the Clinical Research Networks for Rare Diseases Task Force from IRDiRC, specifically regarding the acceleration of adoption and development of widespread (international) new diagnostic tools and therapies, as well as the establishment of findable, usable protocols for data collection, infrastructures, data repository, longitudinal studies, clinical trials and natural history studies.

In addition, the need for a full understanding of disease molecular bases and empowerment of preclinical and translational research, with sound investments, were already highlighted by the European Strategy Forum on Research Infrastructures landmark analysis [34].

The data gathered in 2023 with the third edition of the survey highlighted that the endorsement through the NP/NS for RD to the promotion of the rapid translation of research results in clinical studies and healthcare existed in 38% of the countries, and in 12% to the development of innovative methodologies tailored for clinical trials in RD. Other initiatives for the promotion of the rapid translation of research results in clinical studies and healthcare have been reported by 12% of the countries. For this edition of the survey, it has further been asked whether other initiatives promoted the development of innovative methodologies tailored for clinical trials in RD, with 4% of the countries answering in the affirmative.

The paediatric population deserves special consideration in trials and drug development. The needs of the paediatric population have been extracted from the EPTRI objectives, with the need to accelerate paediatric drug development along the most relevant technological innovations being one of the relevant needs identified.

In the last summary of the mapping of R&I needs series, the barriers to advancement on drug development for children constitute a group of several needs, including the fast translation of research into clinical care. There is a need for children-specific innovative study design applications to translate R&I into specific paediatric and orphan therapeutic approaches, also covering all paediatric ages. The need to increase preclinical information on the basis of innovative tools such as molecular targets, paediatric cellular line models, biomarker variability or animal juvenile models was also raised [35].

A final question posed in the last version of the survey asked about the impacts (promotion, support or triggering) of the EJP RD activities on the countries’ undertakings in three different areas: undertakings that were not deemed or implemented earlier in the country, establishment and/or implementation of data repositories and tools for RD research and the establishment of RD training activities.

A total of 64% of the countries answered that the EJP RD had an impact on the promotion, triggering or helping to enforce RD undertakings that were not deemed or implemented earlier in the country. The areas in which this impact was reflected were, in descending order of frequency: “increased participation in transnational calls for research projects” (69%), “increased participation in national calls for research projects” (38%), “support to the implementation of FAIR data” (31%), “promotion of rapid translation of research results in clinical studies and healthcare” (19%) and “promotion of the development of innovative methodologies tailored for clinical trials” (13%).

Further, 36% of the countries replied that the EJP RD had an impact on the promotion, triggering or helping in the establishment and/or implementation of data repositories and tools for RD research, and namely in the following areas (in descending order of frequency): “registries” (89%), “biobanks catalogue” (78%), “ontologies and codification” (67%), “support to clinical/translational research” (44%), “data deposition and analysis” (44%), “tools” (33%), “access & privacy control” (22%) and “semantic standards” (22%). No country indicated “OMIC services”, “cell lines” or “animal models” as areas of impact.

Finally, 48% of the countries reported an impact of the EJP RD on the promotion, triggering or help in the establishment of RD training activities. The impact on the training activities appeared in the following areas, (in descending order of frequency): “online education courses” (83%), “empowerment of the patients” (67%), “registries” (58%), “data management” (42%), “data quality” (42%), “biobanks” (42%), “FAIR data” (33%) and “standards and quality of genetics/genomics data in clinical practice and laboratories” (25%).

The countries were moreover asked to describe the most significant changes in the RD area occurred since 2019, the starting year of the EJP RD, and 7 of the 19 European countries that replied to the survey reported activities related to the ERNs. Other highlighted topics were: sharing of knowledge on RD and tools for RD; enhancement in diagnosis; widening of newborn screening; enhancements at policy and legal level; increased access to orphan drugs; improvements related to patients organizations; creation on national centres for RD; patient therapeutic education; implementation of measures; wider participation in RD transnational projects; social and psychological tools; hotlines; regulations on telemedicine; increased involvement in NMGs; registries; and genetic testing.

It turned out that 30% of the NP/NS for RD of the EU13 countries promoted the rapid translation of research results in clinical studies and healthcare, and 20% the development of innovative methodologies tailored for clinical trials, whereas other activities for the rapid translation of research results in clinical studies and healthcare have not been reported by any of the EU13 countries.

As previously stated, Table 2 provides the results of the first two editions of the survey, broken down by pillar. It also includes a column dedicated to the EU13 outcomes.

Obstacles and barriers were a specific issue examined by the survey in the EU13 context. The survey investigated obstacles and barriers to development, improvement and translation of RD research results, as well as the participation in EU/international projects in the RD field, with two questions dedicated exclusively to these countries (Table 3). The needs of this domain are very similar to those mentioned for the EU in general, particularly the acceleration of research translation and the development of innovative methodologies for clinical studies.

**Table 3:** Obstacles and barriers specific to EU13 countries

Question		Year		
Topic	Barrier	% detected in 2020	% detected in 2021	% detected in 2023
Development, improvement and translation of RD research results	Funding	83%	89%	90%
	Difficulties in accessing to national resources for funding of research and development of RD projects	50%	56%	80%
	Lack of options for exploitation of research results at national level	50%	40%	20%
	Language	17%	11%	0%
Participation in EU/international projects in the RD field	Limited links to potential partners	100%	78%	40%
	Lack of information on funding opportunities	67%	56%	40%
	Bureaucratic application on responding procedures	50%	50%	80%
	Quality of support provided by national contact points	50%	44%	50%
	Limited skills on drafting proposals	33%	22%	–
	Irrelevance of programme topics and goals to own research agenda	17%	22%	10%

*% detected* % of countries finding the barrier present, *–* not asked that year, *RD* rare diseases

#### Follow-up comparison of the answers submitted by the countries in 2023 versus 2020/2021

The 2020 and 2021 editions of the survey have been considered as one single outcome, given the short time periods between the two data collections, which led to the assumption that there were no significant changes during this period. Nevertheless, the countries that replied in 2020 had the possibility to furnish eventual updates in 2021.

A total of 19 countries were analysed for this follow-up comparison (Table 4). For 15 of the 19 countries (79%) the survey was filled by the same reference person over time, and for other 2 countries by different people belonging to the same institution.

**Table 4:** Status of NP/NS for RD

Existence of a NP/NS for RD	2020/2021	2023
NP/NS for RD active, expired or under renewal	15 countries: Austria, Bulgaria, Czechia, France, Georgia, Germany, Italy, Lithuania, Luxembourg, Portugal, Romania, Serbia, Slovakia, Spain, the Netherlands (79%)	16 countries: Austria, Bulgaria, Czechia, France, Georgia, Germany, Italy, Lithuania, Luxembourg, Poland, Portugal, Romania, Serbia, Slovakia, Spain, the Netherlands (84%)
NP/NS under development	4 countries: Canada, Georgia, Israel, Poland (21%)	2 countries: Canada, Georgia (11%)
No NP/NS for RD	–	1 country: Israel (5%)

Source: Adapted from EJP RD dissemination materials (deliverables)

Of the 19 countries, 84% appeared to have adopted NP/NS for RD at some stage, with an increase of 5% in 2023 in respect to the period 2020/2021. One country that was developing a NP/NS for RD in the period 2020/2021 declared in 2023 not to be provided by a NP/NS for RD, and that its NP/NS for RD was neither active nor under development, indicating a possible halt in the process.

Six of the NP/NS for RD (or list of measures in two cases) that in 2020/2021 were expired appeared in 2023 to be renewed or under renewal.

The follow-up comparisons analysed the variations in the replies submitted by the countries over time in respect to the main items, investigating the alignment status of the NP/NS for RD and of other national RD activities with the four EJP RD pillars.

The considered variations (falling in different areas of each Pillar) are as follows:

i. *Positive variation:* the adoption of undertakings in countries that previously (2020/2021) declared to not have provided initiatives in the field, or to not be aware of their existence.
ii. *Negative variation:* the withdrawal of support to specific initiatives in countries that

formerly declared to endorse the same kind of support.

iii. *Unvaried positive:* the persistence of the promotion of the initiatives over time.
iv. *Unvaried negative:* the persistence of lack of support over the years.
v. *Missing:* lack of information on the topic.

Tables 5 and 6 summarize the variations registered for each relevant topic of the survey. There are no indications on the statistical significance of these variations.

**Table 5:** Comparison considering the variations in the provisions of the NP/NS for RD of the 19 countries

	Pillar 1			Pillar 2			Pillar 3	Pillar 4	
Provisions of the NP/NS for RD	National calls	Transnational calls	Investments to share knowledge	Data repositories and tools	FAIR data	Multidisciplinary holistic approaches	Trainings	Rapid translation of research results	Innovative methodologies tailored for clinical trials
Positive variation	32%	21%	11%	16%	26%	11%	16%	21%	5%
Unvaried positive	26%	32%	21%	42%	11%	68%	63%	21%	/
Unvaried negative	32%	32%	53%	5%	47%	5%	5%	37%	73%
Negative variation	5%	10%	11%	26%	5%	5%	5%	11%	11%
Missing	5%	5%	5%	11%	11%	11%	11%	11%	11%

% = proportion of countries with the result; source: adapted from EJP RD dissemination materials (deliverables); pillar 1: funding, pillar 2: data; pillar 3: capacity-building; pillar 4: translation acceleration

**Table 6:** Comparison considering the variations in the provisions of the other initiatives of the 19 countries

	Pillar 1		Pillar 2				Pillar 3	Pillar 4
Other initiatives	Public funding for research/networking	Private funding for research/networking^¥^	Public initiatives for data repositories and tools	Private initiatives for data repositories and tools	FAIR data	Multidisciplinary holistic approaches	Trainings	Rapid translation of research results
Positive variation	5%	5%	/	11%	11%	5%	5%	5%
Unvaried positive	21%	26%	26%	16%	11%	21%	26%	11%
Unvaried negative	32%	47%	58%	68%	52%	53%	37%	73%
Negative variation	42%	21%	16%	5%	26%	21%	32%	11%
Missing	/	/	/	/	/	/	/	/

^¥^Total 100 without rounding; % = proportion of countries with the result; source: adapted from EJP RD dissemination materials (deliverables); pillar 1: funding, pillar 2: data; pillar 3: capacity-building; pillar 4: translation acceleration

The tables present the observed variations with reference to the NP/NS for RD and to other RD initiatives for all 19 countries, and separately, Tables 7 and 8 for the EU13 countries.

**Table 7:** Comparison considering the variations in the provisions of the NP/NS for RD of the EU13 countries

	Pillar 1			Pillar 2			Pillar 3	Pillar 4	
NP/NS for RD	National calls	Transnational calls	Investments to share knowledge	Data repositories and tools	FAIR data	Multidisciplinary holistic approaches	Trainings	Rapid translation of research results	Innovative methodologies tailored for clinical trials
Positive variation	43%	29%	14%	43%	57%	29%	29%	14%	14%
Unvaried positive	14%	29%	14%	43%	/	57%	71%	14%	72%
Unvaried negative	43%	/	58%	/	/	/	/	58%	/
Negative variation	/	42%	14%	14%	43%	14%	/	14%	14%
Missing	/	/	/	/	/	/	/	/	/

% = proportion of countries with the result; source: adapted from EJP RD dissemination materials (deliverables); pillar 1: funding, pillar 2: data; pillar 3: capacity-building; pillar 4: translation acceleration

**Table 8:** Comparison considering the variations in the provisions of the other initiatives of the EU13 countries

	Pillar 1		Pillar 2				Pillar 3	Pillar 4
Other initiatives	Public funding for research/ networking	Private funding for research/ networking	Public initiatives for data repositories and tools	Private initiatives for data repositories and tools	FAIR data	Multidisciplinary holistic approaches	Trainings	Rapid translation of research results
Positive variation	/	14%	/	/	14%	/	14%	/
Unvaried positive	14%	/	14%	14%	/	43%	14%	/
Unvaried negative	29%	72%	/	72%	72%	14%	29%	100%
Negative variation	57%	14%	86%	14%	14%	43%	43%	/
Missing	/	/	/	/	/	/	/	/

% = proportion of countries with the result; source: adapted from EJP RD dissemination materials (deliverables); pillar 1: funding, pillar 2: data; pillar 3: capacity-building; pillar 4: translation acceleration

As for the comparison of the questions dedicated exclusively to the EU13 countries, the following results have been observed: regarding the participation to EU/international projects, there have been improvements regarding the “link to potential partners”, as well as on the “information on funding opportunities” (positive variation = 38%) and in the perception of “irrelevance of programme topics and goals for the own research agenda” (no longer perceived as obstacles by 57%, 38% and 15% of the countries).

Further, there have been more countries indicating as obstacles “bureaucratic application on responding procedures” (negative variation = 29%) and “quality of support of national contact points” (negative variation = 14%).

Regarding the main perceived obstacles and barriers to development, improvements and translation of RD research results in the EU13 countries have been observed in “options of exploitation of research results at national level” (positive variation = 15%) and “language”, which was no longer indicated as an obstacle by any of the countries.

The topics for which an increase of criticality has been registered were “funding”, indicated by all responding countries (negative variation = 17%), and “difficulties in accessing to national resources for funding of research and development of RD projects” (negative variation = 28%).

### Strategic workshops with national policymakers

The first strategic meeting took place as an online event on 8 July 2021 and represented one of the major activities regarding the translation and impact of prioritization on national and EU strategies.

The meeting highlighted the importance of collaboration and creation of synergies both at the national and EU levels and of the sharing of knowledge. In this perspective, the relevance of the NMGs to increase the countries’ capabilities to integrate into the European RD community was stressed [17].

The second strategic workshop was conducted in hybrid format in Brussels on 5 July 2023. A specific focus was placed on EU13 countries, as facing challenges related to financing, infrastructure, drug reimbursement and facing underrepresentation in EU programmes, particularly in leadership roles.

Although facing significant challenges, EU13 MS showed great opportunities, with the development of new medicinal products or artificial intelligence (AI) [36, 37].

The workshop concluded with the issuing of several recommendations and the recognition of the potential of multicentre clinical trials and AI tools.

Two detailed reports on the meetings and on the actions proposed to promote the implementation of successful and targeted RD actions have been published [17, 38].

### R&I needs addressed by EJP RD activities over the years

As seen in the methods section, the IRDiRC’s goals were utilized to group all the mapped needs. EJP RD planned its annual activities to address them. The complete list of needs classified by IRDiRC goal and domain is presented in Additional file 2: “Compilation of mapped needs on R&I”. The list of covered needs can be consulted in Additional file 3: “Mapped needs covered by EJP RD”.

We created a monitoring indicator to evaluate the needs covered by the Programme. This indicator calculated the ratio of the number of activities carried out by EJP RD to the total number of needs identified during the mapping process (percentage of needs covered = number of EJPvRD activities/total needs listed). We obtained a global coverage of 65.8%, with greater coverage of needs for IRDiRC goals 1 and 2. The summary of results are presented in Table 9.

**Table 9:** Summary analysis of alignment R&I needs and EJP RD activities

Paths	Total needs listed	Number activities carried out by EJP RD (*n*)	Percentage of needs covered by EJP RD
Diagnostic path (IRDIRC goal 1)	28	20	71%
Treatment path (IRDIRC goal 2)	14	10	71%
Both paths interconnected (IRDIRC goals 1–2)	5	5	100%
Methodologies (IRDIRC goal 3)	18	8.5	47%
Transversal/ nonscientific domains (diagnosis & healthcare; regulatory& ethics; EU competitiveness & innovation)	36	23	64%
Total	101	66.5	65.8%

Source: adapted from internal EJP RD materials

### National Mirror Groups: bridging national and European rare diseases efforts

In the pursuit of facilitating coordinated action and policy alignment in rare disease research, and to respond to the needs identified through the activities of the WP2, the EJP RD has spearheaded the establishment of NMG across beneficiary countries, designed to bridge the gap between national RD efforts and overarching European strategies. This was one of the most pressing needs identified by the project and is therefore detailed in this separate section.

EJP RD’s consortium, comprising diverse partners and stakeholders, has underscored the significance of leveraging existing resources, identifying weaknesses, and innovating novel solutions to address RD challenges. Central to this endeavour is the strategic engagement of policymakers at regional, national, EU and international levels.

The implementation of NMG has been initiated under the EJP RD lifetime to facilitate the gathering of RD stakeholders at the national level and to form a link with activities and plans being delivered by large European consortia dedicated to RD.

A NMG is a group created – or designated – in each country benefitting from the EJP RD to bring together the expertise and knowledge of the RD community of a specific country.

The objectives of NMGs are, on one hand, to identify national needs that should be discussed and addressed, if possible, within the framework of the EJP RD activities, and on the other hand, to promote national alignment with the European RD research strategy. In addition, the lessons learned and good practices collected by each country are shared with the others. In this way, the activities of the EJP RD are more readily informed and shaped by heterogeneous national needs and realities, whilst the tools, assets and approaches developed under the EJP RD are implemented more at the national level to advance RD research and innovation for all stakeholders. Additionally, NMGs can play a crucial role in advocating for necessary policy changes or improvements. By bringing together influential stakeholders and experts, NMGs can collectively identify policy gaps and advocate for reforms that would create a more favourable environment for the project’s success. They can advocate for necessary policy changes to create an enabling environment for the project’s success.

The added value of NMG has been identified through past projects and initiatives, in particular those highlighting the need to revive national commitments to RD. The first foresight study for RDs, Rare 2030, identified the fundamental need for “a new EU policy framework for RD guaranteeing that RDs remain a public health priority through concerted European actions and guiding the implementation of long-term national plans and policies across all countries in Europe”. Rare 2030 also included several recommendations specifically around the need for greater connectivity and alignment of what happens at the national and European levels, with enhanced opportunity for identifying and sharing good practices which are being employed in some places and could be translated to other national settings. The final project recommendations specifically mention the need to create more NMGs as a way to achieve this greater alignment. This is viewed as an important means of stimulating renewed national focus on maintaining dynamic and robust national plans for RD, which has proved challenging for many European countries. NMGs also hold great potential to align national activities with the IRDiRC goals.

During the project’s tenure, substantial progress was made in the development of NMGs: four were developed during the 5 first years of the EJP RD in France, the Netherlands, Portugal and the United Kingdom. In 2023, significant progress was accomplished for the development of Rare Diseases NMG, especially through the development of the European Rare Diseases Research Alliance Partnership proposal. Building upon activities such as national meetings and survey analyses from the previous year, fresh connections were established with EJP RD partners. These connections were instrumental in preparing for the development of NMG. The WP2 coordination team gained insights into the situation across more than 25 countries and initiated contacts with organizations already engaged in national groups. They have also reached out to representatives of countries and organizations eager to establish such groups, possessing extensive knowledge of their respective rare disease communities.

## Discussion

The comparisons along the surveys highlight the efforts required to align RD research and innovation strategies across Europe and other countries. Although variations between respondents and responses from countries in the different surveys may limit the interpretation of the results, we have obtained a global picture of the landscape of national policies and actions for rare diseases over the years.

With respect to the “national and International investments on research to field of RD”, investments to share knowledge required particular attention, especially in EU13 countries. In addition, further efforts were advisable to maintain and improve the positive outcomes observed in the promotion of national and transnational calls for research projects. This observation concerned both the NP/NS for RD and the other RD initiatives.

As for “resources and services to foster research on RD”, an overall positive alignment of the NP/NS for RD has been highlighted, particularly in the EU13 countries, whilst such positive outcome did not appear regarding the other initiatives. A special effort emerged to further enhance the support to FAIR data by the NP/NS for RD and by other initiatives.

Looking at “capacity-building and empowerment”, more attention to RD trainings by initiatives other than the NP/NS for RD emerged as being needed as well.

Finally, considering the “accelerated translation of research projects and improvement of outcomes of clinical studies”, both the rapid translation of research results in clinical studies and healthcare and the development of innovative methodologies tailored for clinical trials were raised as areas that required high attention, particularly in EU13 countries.

Additionally, the trend in R&I needs throughout the programme highlights fundamental issues that need to be addressed on an ongoing basis.

A renewal of the policy framework for rare diseases at the European level is urgently needed. There is a demand from the RD community for an action plan for RD, which should support and feed into national RD strategies and plans [32]. This policy framework should be accompanied by long-term sustainable investment in the European RD research ecosystem.

Basic, clinical, social and translational research on rare diseases should be maintained as a priority, with an increase in research to support the development of innovations for research on treatments and therapies. Diagnosis is crucial, and the need to facilitate and widen access to scientific advances (e.g. next-generation sequencing techniques, imaging, artificial intelligence and other digital solutions, in addition to improving screening programmes), has been mapped over the years, especially regarding timely diagnosis and disregarded rare diseases. Research, diagnosis, treatment and care need to be integrated along the same path, that is, with a holistic vision, as has been shown in previous years. The needs for advances in treatment (e.g. repurposing of drugs, discovery of molecular targets or biomarkers) is particularly relevant for the paediatric population, which requires special attention in terms of addressing unmet needs [35].

Data strategies, especially for the ERNs, are a R&I priority. The need to facilitate and improve data sharing in health research is a constant in the identification of R&I needs. This need is linked to the need to accelerate the translation of research results into clinical studies and healthcare and to improve the outcomes of clinical studies. In detailing data needs, there is a priority for data standardization and safety (i.e. authorization and authentication) and privacy (preserving issues), including harmonization, interoperability and patient involvement in data collection with FAIR principles. Data sharing and reuse, particularly with data generated from studies (e.g. clinical trials) appears to be an important need. There is also a need to optimize electronic health records across Europe whilst linking ERNs to national health systems. Priority areas to focus on are the new challenges of data and digital health technologies, as well as a holistic lifelong approach and social inclusion.

Training and capacity-building, including career development and gender balance, as well as stakeholder literacy, especially on health data rights, remained a need in all mappings.

Collaboration and integration with other infrastructures, such as the European Health Data Space (EHDS), the European Alliance of Medical Research Infrastructures (EU-AMRI)—comprising the Medical Research Infrastructures EATRIS-ERIC, focussed on translational medicine, ECRIN-ERIC, focussed on multinational clinical research, and BBMRI-ERIC, focussed on biobanking and biomolecular resources—sustains and also generates R&I needs that permeate the entire ecosystem.

Special considerations outside of the topics mentioned above are related to the global context of the last few years. It can be said that, despite some progress, the diagnosis and treatment of rare diseases have been negatively affected in recent years as a result of the COVID-19 pandemic, which has aggravated the difficult situation of healthcare, diagnosis, treatment and research for patients with rare diseases [39].

In addition, the direct consequences of the war context in Ukraine have also contributed to debilitating an already vulnerable population, increasing the difficulties faced by the RD community there and in neighbouring countries, including EU13 countries such as Poland and Romania [40]. Medium-term access to secure and disability-adapted housing, access to basic services, psychological support, medications and supplies, equipment and hospital beds, the need for the involvement of the international community and the need for tackling language barriers were amongst the most relevant [41–43]. Regarding the last point, the need for overcoming language barriers (mentioned through the years) has been generally identified, not only in the war context.

As an expected impact, EJP RD aimed to improve the alignment of national/regional activities and policies in RD. The strength of the EJP RD consortium lay not only in the number and diversity of its partners, but also in its capacity to exploit the existing elements, identify their weaknesses, improve them, innovate and deliver new, more efficient solutions. To efficiently transcribe EJP RD activities and outcomes at the regional, national, EU and international levels, the EJP RD was in connection with relevant policymakers. The governance, research and innovation strategy and prioritization process involved the Policy Board, with ministries of research and health, relevant EC directorates and other key high-level stakeholders. In addition, the NMGs seek to include representatives of national strategies for RD, national nodes of the ERNs, relevant national authorities and research institutions (whether participating in the EJP RD or not) that help the alignment of the integrative research and innovation strategy for rare diseases.

## Conclusions and future

RD research provides many opportunities for research and public health, which should be taken advantage of to foster benefits for patients, healthcare systems and the scientific community, but at the same time there are various challenges to be addressed [44]. Indeed, several needs identified throughout the project need further approach. There are barriers related to social justice, advances in personalized medicine and provision of healthcare for patients with rare diseases. Increasing the availability and accessibility to drugs (including the facilitation of the regulatory pathway for orphan products authorization) and medical devices is a relevant need to reduce inequality across Europe. Adoption and implementation of real-world evidence in all the steps would help this accessibility as well. Optimization of data and innovative technologies still represent a need that lacks alignment. A policy framework that encompasses EU and its MS has been pursued [33].

Needs for diagnosis remain present as well. The secure pooling of genomic data and biosamples in collaborative infrastructures, the search for outcomes complementary to clinical information or the accessibility of underserved populations to RD diagnostics are some of the needs that need more alignment. Completing these we can find the improvement of diagnostic tools and genetic methods, imaging AI or other digital solutions.

Including assisted and daily life technologies and research on complex therapeutic targets and innovative therapies such as advanced therapies, gene editing or cell therapies are notable amongst the treatment needs that require attention.

There is a need for greater alignment on certain methodological advances, highlighting the need for broad strategy trial design, computational model development, artificial intelligence, big data and block or cost-effective innovative methods for drug development.

Further, there is the necessity to keep the momentum on the importance of active and up-to-date NP/NS for RD, as recommended by the Commission Communication and Council Recommendation [45, 46], as well as the necessity to foster the establishment of the NMGs, including representatives of each NP/NS for RD, national nodes of the ERNs, relevant national authorities and research institutions. In this direction, the constitution of dedicated RD advisory bodies for Research and Innovation for RD should also be considered.

Further promotion of research activities is required, particularly in the form of national calls for research projects, alongside investments to share knowledge on RD.

Moreover, the adoption of FAIR data principles, particularly through the NP/NS for RD and the exploitation of trainings dedicated to FAIR data, have been recognized as priorities, together with the boosting of all activities related to the accelerated translation of research projects and improvement of outcomes of clinical studies, namely the rapid translation of research results into clinical studies and healthcare and the development of innovative methodologies tailored for clinical trials.

As to the specific needs of EU13 countries, there is a demand to enhance participation in national and transnational calls in research projects to foster the adoption of FAIR data by the NP/NS for RD and to improve the promotion of the translation of research results into clinical studies and healthcare as well as the development of innovative methodologies tailored for clinical trials. In more detail, for these countries specific requirements include increasing RD-dedicated funding, facilitating access to funding for research and development of RD projects, enlarging the possibilities to exploit research results at national level, facilitating the links to potential partners, easing the retrieval of information on funding opportunities, assisting on bureaucratic application on funding procedures and improving the quality of support provided by the national contact points.

The next years will be fundamental for the future coverage of all the identified needs and for the sustainability of all work done thus far, taking into account the coordination and alignment of funding and national actions, the innovation in the translation of research results and the reach of a holistic R&I ecosystem all in all, without leaving behind the nonscientific domains (diagnosis and healthcare, regulation and ethics and competitiveness and innovation).

Regarding NMGs, the ongoing effort helped establish seven new NMGs by mid-2024, which was a significant milestone towards achieving this objective by the end of the EJP RD project. The following countries had operational NMGs by the end of the EJP RD: Austria, Canada, Cyprus, Estonia, France, Lithuania, the Netherlands, Portugal, Slovakia, Turkey and the United Kingdom. Some additional countries continued the process of establishing theirs.

The transition to the European Rare Diseases Research Alliance (ERDERA) marked a new chapter in the evolution of NMGs as well. The goal of ERDERA is to increase the number of NMGs, with plans to have 37 by the first year and a half. Building on EJP RD’s groundwork, ERDERA inherits the mandate to fortify NMGs’ role in fostering collaboration, sharing best practices and advocating for policy reforms. During the first year of ERDERA, 6 new NMGs have been developed (Belgium, Ireland, Latvia, Serbia, Spain and Sweden) and a further 13 are currently in the process of being developed (Australia, Bulgaria, Czechia, Denmark, Greece, Italy, Luxembourg, Morocco, New Zealand, Norway, Poland, Slovenia and Switzerland). The imperative for greater connectivity and alignment, as emphasized in initiatives such as Rare 2030, underscores the pivotal role of NMGs in harmonizing national and European RD agendas.

ERDERA’s agenda underscores a dual-pronged approach: bolstering existing NMGs and expanding their footprint across beneficiary countries. The formation of NMGs in countries lacking national working groups on RDs represents a critical stride towards comprehensive RD policy alignment. Moreover, adapting existing national groups to align with ERDERA’s objectives ensures a cohesive and synergistic approach towards addressing RD challenges.

## Supplementary Information

Additional file 1.“Original Survey”: PDF containing the original survey to Member States, which has been updated over the years

Additional file 2. “Compilation of mapped needs on R&I”: PDF containing two boxes, one compilation of all mapped needs on R&I during the EJP RD (diagnosis, treatment and other with the related domains (transversal) mapped needs)

Additional file 3. “Mapped needs covered by EJP RD”: PDF containing the list of mapped needs covered by EJP RD activities

## Data Availability

The data from surveys and the alignment of research innovation needs mapping with the activities of the programme is provided within the manuscript. Additional data from the surveys can be found on the website of the EJP RD, deliverables section: https://www.ejprarediseases.org/our-publications/public-deliverables-2/ Additional data from research and innovation needs mapping is provided in the supplementary information files.

## Abbreviations

AI: Artificial intelligence
EJP RD: European Joint Programme on Rare Diseases
ERDERA: European Rare Diseases Research Alliance
ERN: European Reference Network
FAIR: Findable, accessible, interoperable, reusable (data for humans and machines)
GDPR: General data protection regulation
IRDiRC: International Rare Diseases Research Consortium
JTC: Joint transnational calls
MS: Member states
NMG: National mirror groups
NP/NS: National plans/national strategies
R&I: Research and innovation
RD: Rare diseases
SME: Small and medium enterprises
WP: Work package

## Footnotes

1 Activity led by Institute for Rare Diseases Research (IIER) from Instituto de Salud Carlos III (ISCIII), Spain.

2 European Rare Disease Research Coordination and Support Action Consortium (ERICA) for ERN information, European Strategy Forum on Research Infrastructures (ESFRI roadmap), European Paediatric Translational Research Infrastructure (EPTRI).

3 Activity led by Istituto Superiore di Sanità (ISS), Italy.

4 Refers to countries that joined the EU after 2004 (Bulgaria, Croatia, Cyprus, Czechia, Estonia, Hungary, Latvia, Lithuania, Malta, Poland, Romania, Slovakia and Slovenia). EU13 Member States are also known as widening countries https://ec.europa.eu/newsroom/jrcseville/items/56531/en

5 Activity carried out by Institut National de la Santé et de la Recherche Médicale (INSERM), France.

6 One Strategic Workshop organized by ISS, Italy, and other by Medical University of Warsaw (MUW), Poland.

7 No information was obtained from Greece and Finland.

8 Armenia, Belgium, Croatia, Georgia, Latvia and Poland.

9 Austria, Bulgaria, Canada, Cyprus, Czechia, Denmark, Estonia, France, Georgia, Germany, Israel, Italy, Lithuania, Luxembourg, Malta, Norway, Poland, Portugal, Romania, Serbia, Slovakia, Slovenia, Spain, Switzerland and the Netherlands.

10 Bulgaria, Cyprus, Czechia, Estonia, Lithuania, Malta, Poland, Romania, Slovakia, Slovenia.
