# Supplementary material for: Integrative research and innovation strategy for rare diseases. Insights from the 5-year European joint programme on rare diseases, including analysis to inform recommendations for future actions
Source: Health Res Policy Syst. 2025 Oct 16;23:137. doi: 10.1186/s12961-025-01389-7 (PMC12532414; doi:10.1186/s12961-025-01389-7)
Supplement: Supplementary file 1 — Additional file 1.“Original Survey”: PDF containing the original survey to Member States, which has been updated over the years [file 12961_2025_1389_MOESM1_ESM.pdf]

Integrative Research and Innovation Strategy for Rare Diseases. Insights from the five-year European Joint Programme on Rare Diseases, including analysis to inform recommendations for future actions.

Original survey

National Plans and Strategies for Rare Diseases

Content of the survey

Link to the online survey:
https://sondage.inserm.fr/index.php/638715/lang-en

EUROPEAN JOINT PROGRAMME RARE DISEASES

National Plans and Strategies for Rare Diseases

This survey aims at collecting information from EU Member States on the state of the art regarding the development and implementation of National Plans and Strategies for rare diseases and on the alignment process with the European Joint Programme on Rare Diseases, EJP RD, (GA 825575) relevant/complementary actions performed at national level, with a specific focus on EU 13 Countries in respect to their specific needs, obstacles and advancements.

Please fill in the survey by referring to the National Plan or Strategy for rare diseases in your Country.

Be aware that some questions do not relate directly to the National Plan or Strategy for rare diseases but concern the rare disease field in a broader context.

Estimated time for completing the survey: 45 minutes

Deadline: October 16th

There are 59 questions in this survey.

National Plan/Strategy for rare diseases in your Country 1

Existence, periodical evaluation, update, expiartion of National Plan and Strategy for rare diseases in your Country

*Is there an approved National Plan/Strategy for rare diseases in your Country?
Choose one of the following answers

- (selected) **Yes**
- **Yes but not in force**
- **No**
- **No but it is under development in my Country**
- **I do not know**

If yes, please provide the link to the text of the National Plan/Strategy for rare diseases in your Country

When was the National Plan/Strategy for rare diseases approved in your Country? (specify year)

*
Is there a periodical evaluation of the National Plan/Strategy for rare diseases in your Country?
Choose one of the following answers

- Yes
- No
- I don't have this information

*
By now, how many editions of National Plans/Strategies has your Country adopted ?
Choose one of the following answers

- One
- More than one
- I don't have this information

Involvement in the National Plan/Strategy for rare diseases 1

* Are you directly involved in the development, implementation or review of the National Plan/Strategy for rare diseases in your Country?

- (x) **Yes**
- ( ) **No**

National and International Investments on research in the field of rare diseases 1

* Does the National Plan/Strategy for rare diseases in your Country promote calls for research projects on rare diseases?
Please select at most one answer

- [x] **Yes**
- [ ] **No**
- [ ] **I do not know**

* Please specify how the National Plan/Strategy of your Country promotes national calls for research projects and provide a link to any existing permanent dedicated website/webpage.

National and International Investments on research in the field of rare diseases 2

* Does the National Plan/ Strategy for rare diseases in your Country promote transnational calls for research projects?
Please select at most one answer

- [x] Yes
- [ ] No
- [ ] I do not know

* Specify how the National Plan/Strategy for rare diseases of your Country promotes transnational calls for research projects and please, provide a link to any permanent dedicated website/webpage if it exists for calls.

National and International Investments on research in the field of rare diseases 3

* Does the National Plan/ Strategy for rare diseases in your Country foresee investments for networking to share knowledge on rare diseases?
Please select at most one answer

☑ Yes

☐ No

☐ I do not know

* Please specify how the National Plan/ Strategy for rare diseases foresees investments for networking to share knowledge on rare diseases

National and International Investments on research in the field of rare diseases 4

* Are there public funding initiatives in your Country for research and/or networking in the field of rare diseases?
Please select at most one answer

☑ Yes

☐ No

☐ I do not know

* Please describe the public funding initiatives in your Country for research and/or networking in the field of rare diseases

National and International Investments on research in the field of rare diseases 5

* Are there private funding initiatives in your Country for research and/or networking in the field of rare diseases?

Please select at most one answer

- [x] Yes
- [ ] No
- [ ] I do not know

* Please describe the private funding initiatives in your Country for research and/or networking in the field of rare diseases and provide a link to any permanent dedicated website/webpage if it exists.

Resources & services to foster research on rare diseases 1

*Is there an advisory body of national experts for EU Research and Innovation policy in your Country?
Check any that apply

☐ **Yes, an advisory body exists, but not specific for rare diseases**

☐ **Yes, an advisory body specific for rare diseases exists**

☐ **No**

☐ **I do not know**

*How does the National Plan/Strategy for rare diseases of your Country foresee the support of data repositories and tools in research on rare diseases?
Please select at most one answer

☐ **The National Plan/Strategy for rare diseases does not forsee the support of data repositories and tools in rare diseases research**

☑ **Promoting the implementation of such data repositories and tools**

☐ **Promoting the development of new data repositories and tools**

☐ **Promoting both their implementation and development**

☐ **I do not know**

ℹ Already existing examples: RD-Connect platform, European Rare Disease Registry Infrastructure (ERDRI),Cellosaurus, Infrafrontier, ORDO, HPO, EUPID, DECIPHER, EGA

*

If the National Plan/ Strategy for rare diseases of your Country foresees the support of data repositories and tools in research on rare diseases, please specify on which topics (Please select. Possible multiple choice)

Check any that apply

- ☐ **Registries catalogue**
- ☐ **Biobanks catalogue**
- ☐ **Ontologies and codification**
- ☐ **OMIC services**
- ☐ **Cell lines**
- ☐ **Animal models**
- ☐ **Semantic standards**
- ☐ **Support for clinical/translational research**
- ☐ **Access & privacy control**
- ☐ **Data deposition & analysis**
- ☐ **Tools**
- ☐ **Other:** ____________

Resources & services to foster research on rare diseases 2

Do the National Plan/ Strategy for rare diseases or other initiatives for rare diseases support FAIR* data in your Country?
(*FAIR: Findable, Accessible, Interoperable, Reusable)

Please select at most one answer

- [ ] No National Plan/Strategy for rare diseases and no other iniziatives support FAIR data
- [ ] Yes only the National Plan/Strategy for rare diseases
- [ ] Yes only other initiatives for rare diseases support FAIR data but not the National Plan/Strategy for rare diseases
- [x] Yes, both (National Plan/Straegy for rare diseases and other initiaztives for rare diseases)
- [ ] I do not know

The FAIR Guiding Principles for scientific data management and stewardship: https://www.nature.com/articles/sdata201618

Please describe how the National Plan/Strategy for rare diseases or other initiatives for rare diseases support FAIR data in your Country

Resources & services to foster research on rare diseases 3

* Is the adoption of multidisciplinary holistic approaches for rare diseases diagnostics and therapeutics promoted by the National Plan/ Strategy for rare diseases or by other initiatives for rare diseases of your Country?

Please select at most one answer

- ☐ Not by National Plan/Strategy for rare diseases and not by other initiatives for rare diseases
- ☐ Yes only by the National Plan/Strategy for rare diseases
- ☐ Yes only by other initiatives for rare diseases
- ☑ Yes by both, National Plan/Strategy for rare diseases and by other initiatives foe rare diseases
- ☐ I do not know

* Please describe how the adoption of multidisciplinary holistic approaches for rare diseases diagnostics and therapeutics is promoted by the National Plan/ Strategy for rare diseases or other initiatives for rare diseases of your Country

Resources & services to foster research on rare diseases 4

* Are there other public funding initiatives in your Country than National Plan/Strategy for rare diseases for the development of data repositories and tools to support research on rare diseases?

Please select at most one answer

- [x] Yes
- [ ] No
- [ ] I do not know

* Which topics are covered by other public funding initiatives in your country, other than National Plan/Strategy for rare diseases, for the development of data repositories and tools to support research on rare diseases? (please select. Possible multiple choice)

Check any that apply

- [ ] Registries catalogue
- [ ] Biobanks catalogue
- [ ] Cell lines
- [ ] Animal models
- [ ] Semantic standards
- [ ] Support for clinical/translational research
- [ ] Access & privacy control
- [ ] Data deposition & analysis
- [ ] Tools
- [ ] Other: ________

Resources & services to foster research on RDs 5

*

Are there other private funding initiatives in your Country than National Plan/Strategy for rare diseases, for the development of data repositories and tools to support research on rare diseases?
Please select at most one answer

☑ Yes

☐ No

☐ I do not know

*

Which topics are covered by private funding initiatives in your Country, others than those forseen in the National Plan/Strategy for rare diseases for the development of data repositories and tools to support research on rare diseases? (Please select. Possible multiple choice)
Check any that apply

☐ Registries catalogue

☐ Biobanks catalogue

☐ Cell lines

☐ Animal models

☐ Semantic standards

☐ Support for clinical/translational research

☐ Access & privacy control

☐ Data deposition & analysis

☐ Tools

☐ Other:

Capacity building 1

* Does the National Plan/Strategy for rare diseases promote and/or support training activities?
Please select at most one answer

☑ Yes

☐ No

☐ I do not know

* On which topics does the National Plan/Strategy for rare diseases promote and/or support training activities? (Please specify. Possible multilple chioce)
Check any that apply

☐ Data mangment

☐ Data quality

☐ FAIR data

☐ Standards and quality of genetics/genomics data in clinical practice and laboratories

☐ Registries

☐ Biobanks

☐ Empowerment of the patients

☐ Online education corses

☐ Other: ______

* Please describe more in detail how the National Plan/Strategy for rare diseases in your Country promotes/supports training activities in the field of rare diseases and please, provide a link to any permanent dedicated website/webpage if it exists.

Capacity building 2

*

Apart from the provisions made in the National Plan/Strategy, are there other training, mentoring and coaching activities in the field of rare diseases provided in your Country?

Please select at most one answer

☑ **Yes**

☐ **No**

☐ **I do not know**

*

Please describe the other training, mentoring and coaching activities, apart from the provisions made in the National Plan/Strategy for rare diseases, provided in your Country and please, provide a link to any permanent dedicated website/webpage if it exists.

1 Accelerated translation of research projects and improvement of outcomes of clinical studies

Accelerated translation of research projects and improvement of outcomes of clinical studies in the field of rare diseases

* Does the National Plan/Strategy for rare diseases of your Country promote a rapid translation of the research results in clinical studies and healthcare?

Please select at most one answer

☑ Yes

☐ No

☐ I do not know

* Please specify how the National Plan/Strategy for rare diseases of your Country promotes a rapid translation of the research results in clinical studies and healthcare

2 Accelerated translation of research projects and improvement of outcomes of clinical studies

* Does the National Plan/Strategy for rare diseases of your Country promote the development of innovative methodologies tailored for clinical trials in rare diseases?

Please select at most one answer

- [x] Yes
- [ ] No
- [ ] I do not know

* Please specify how the National Plan/Strategy for rare diseases of your Country promotes the development of innovative methodologies tailored for clinical trials in rare diseases

3 Accelerated translation of research projects and improvement of outcomes of clinical studies

* Are there other initiatives that promote a rapid translation of the research results in clinical studies and healthcare in the field of rare diseases, other than the National Plan/Strategy for rare diseases in your Country?

Please select at most one answer

☑ Yes

☐ No

☐ I do not know

* Please specify what other initiatives in the field of rare diseases promote a rapid translation of the research results in clinical studies and healthcare, other than the National Plan/Strategy for rare diseases in your Country

Additional Information 1

This group of questions is dedicated to EU 13 Countries, with regard to their specific advancements, obstacles and needs in the field of rare diseases

* Do you belong to an EU 13 Country?

- (•) **Yes**
- ( ) **No**

EU 13 countries: Bulgaria, Croatia, Cyprus, Czech Republic, Estonia, Hungary, Latvia, Lithuania, Malta, Poland, Romania, Slovakia and Slovenia

* To which EU 13 Country do you belong?
Choose one of the following answers

Please choose...

* Based on your experience, what are the main obstacles and barrieres in your Country for the development, improvement and translation of rare disease research results ? (Please select. Possible multiple choice)
Check any that apply

- [ ] **Language**
- [ ] **Fundings**
- [ ] **Difficulties in accessing to national resources for funding research and development of RD projects**
- [ ] **Lack of options for exploitation of research results at national level**
- [ ] **Other**

* Please describe more in detail the main obstacles and barrieres in your Country for the development, improvement and translation of rare disease research results, based on your experience

*

Regarding your participation in EU/International projects in the rare diseases field, what do you estimate are the most important obstacles/barrieres ? (Please select. Possible multiple choice)

Check any that apply

- ☐ **Limited skills on drafting proposals**
- ☐ **Lack of information on funding opportunities**
- ☐ **Limited links to potential partners**
- ☐ **Bureaucratic application on reporting procedures**
- ☐ **Irrelevance of programme topics and goals to own research agenda**
- ☐ **Quality of support provided by national contact points**

*

Please describe more in details what you estimate as the most important obstacles/barrieres for the participation of your Country in EU/International projects in the rare diseases field

Additional Information 2

* Do you want to highlight any other aspect regarding the National Plan/Strategy for rare diseases of your Country that was not included in the present survey?

- (●) Yes
- ( ) No

if Yes please specify

Source: Original work from the Consortium WP2
