# Supplementary material for: Integrative research and innovation strategy for rare diseases. Insights from the 5-year European joint programme on rare diseases, including analysis to inform recommendations for future actions
Source: Health Res Policy Syst. 2025 Oct 16;23:137. doi: 10.1186/s12961-025-01389-7 (PMC12532414; doi:10.1186/s12961-025-01389-7)
Supplement: Supplementary file 2 — Additional file 2. “Compilation of mapped needs on R&I”: PDF containing two boxes, one compilation of all mapped needs on R&I during the EJP RD (diagnosis, treatment and other with the related domains (transversal) mapped needs) [file 12961_2025_1389_MOESM2_ESM.pdf]

Box 1: Compilation of mapped needs on Research and Innovation

EJP RD DIAGNOSIS PATH & IRDIRC'S GOAL 1	BOTH PATHS<br>INTERCONNECTED (applicable to both)	EJP RD TREATMENT PATH<br>& IRDIRC'S GOAL 2
• Easier and more generalized automated exchange of phenotypic data.<br>• Further development of standards and interoperability for data and resources.<br>• Accessible and useful workflows/pipelines for RD data analysis.<br>• Collection and linking of high-quality data and biological samples between biobanks and registries (e.g. unique identifiers for patients).<br>• Upgrade of natural history studies, disease progression and mechanisms, including biomarkers.<br>• Identification of clinical biomarkers, clinical outcome measures and surrogate endpoints.<br>• Complement data entries by clinicians by patients themselves (PROMs).<br>• Linking registries and databases to Centers of Expertise.<br>• Improvement and development of diagnostic tests, including better	• ***QUALITY:*** Harmonising procedures; harmonised quality requirements for registries and biorepositories; optimisation and innovation in Information & Comm Tech, e-infrastructures, open e-networks, virtual biobanks; establish findable, usable protocols for data collection, infrastructures, data repositories; longitudinal studies, clinical trials and natural history studies, tools and common goals.<br>• ***SUSTAINABILITY:*** Resources optimization and sharing (multipurpose registries, cluster registries, epidemiological platform and infrastructures); compromises on public funding, e.g. European funding of health networks; sustainable economic models for development and commercialization of orphan drugs.<br>• ***PATIENT EMPOWERMENT:*** Innovative ways for engagement in drug development and clinical trials; patients' needs driven research: better capture of patients' needs; holistic patient-centred approach, inclusion in governance and decision-making setting; capacity building and awareness campaign; options on raising funds for research on their disease and leading their own research projects.	• Stimulation of novel therapies development through the understanding of the genetic basis, molecular and pathophysiological mechanisms and the natural history of RD.<br>• Research into combining therapeutic agents, given the complex pathophysiological mechanisms of RD.<br>• Optimisation of research on preclinical settings, including proof of concept studies of new therapies, validation of disease models and biomarkers.<br>• Innovative approaches in clinical trial design (adaptive designs, small sizes, clinical end points, repurposing, natural history studies, and precision medicine results, patient-centered, with identification of appropriate biomarkers and surrogate endpoints.<br>• Support to clinical research and registries, at a national and international level through funding, facilities to find patients and patients' registries, biobanks and software.<br>• Framework for basket trials of drugs targeting shared molecular etiologies.

understanding of variants underlying phenotypic difference.<br>• Identification of novel pathophysiological pathways in appropriate disease and animal models that effectively mimic the human condition.<br>• Secure pooling of genomic data in collaborative networks and infrastructures, also ensuring the collection and storage of samples in appropriate biorepositories (quality and standards for samples).<br>• Improved annotation and interpretation of variants and development of diagnostic tests for the more prevalent variants.<br>• Novel modalities of functional analysis of candidate variants through in vitro, cell, tissue or animal studies.<br>• Increase the comprehension of the noncoding regions of the genome.<br>• Optimisation and complementarity of WES, WGS, transcriptome sequencing and long read technologies.<br>• Safe deposit of omics data in open or controlled access resources.<br>• Increase of the multi-omics expertise.	• ***DATA***: Comprehensive and integral health data strategy (e.g. data governance, stewardship) in ERNs & DS platforms; Innovative uses for data, RWD, AI & big data. Improvement of data-linkage from multiple sources and settings; enhancement of data quality, FAIRness, connectivity, interoperability, with common data sets and ontologies.<br>• ***NETWORKS, COLLABORATIVE ACTIONS:*** Inclusive pan-European multi-stakeholder networks of EU research, patient and healthcare organizations; clinical research networks; collaboration with ERNs on national integration; Open Science (open access to publications, data, and to research data management plans); international framework of collaboration on best practices; IRDIRC global collaboration. Make easier to find suitable facilities or expertise for research (e.g. multi-omics expertise), biobanks/bio samples/cell lines & patients. Capacity building to increase the knowledge on existing resources and general understanding on standards and data FAIRification concepts; globally coordinated diagnostic and research pipeline; cooperation at a large scale (undiagnosed patients); social media networks opportunities; enabling of research approaches that combine genetics, environmental and societal challenges; overcome	• Scientific research on the role of surgery and/or complementary treatments within a broader strategy of care for RD.<br>• Adapt implementation of regulatory requirements, especially for clinical trials in RD.<br>• Faster uptake of new technologies, within a delivery ecosystem for RD therapies, including assisted and daily life technologies.<br>• Research on complex therapeutic targets and innovative therapies such advanced therapies-gene editing, cell therapies.<br>• Acceleration of the research translation into therapies for patients.<br>• Earlier research and development of treatments for extremely rare diseases.<br>• Creation of guidance and tools on drug repurposing.<br>• Evidence generation for therapies for the very and extremely rare diseases.

• Omic or multi-omic integrated approaches for discovery of disease causes and mechanisms including development of relevant bioinformatic tools.<br>• New schemes for finding diagnosis for undiagnosed patients, and diagnosis accelerators.<br>• Development and timely adoption of new diagnostic technologies and tools.<br>• Improve access to and utility of rare disease diagnostics in underserved populations.<br>• Screening programs enhancement.<br>• Accessible and coordinated diagnostic ecosystem, including healthcare system.<br>• Facilitate and expand access to imaging, artificial intelligence, and other digital solutions to speed up diagnosis.<br>• Phenotype-driven diagnosis: integration across different ontologies, integration of shared pathways, digital phenotyping, development of artificial intelligence approaches/applications to extract health related data in aid of diagnosis.<br>• Functional strategies to globally stratify variants of unknown significance (VUS) for clinical use; setting up of (in vitro) systems	the language barriers, and the lack of a critical mass and role of RD patients (specially EU-13).	

to distinguish between VUS and pathogenic variants (e.g., confirming disruption of splicing for deep intronic variants, loss of protein function, and gain of toxic protein function).		

- ***METHODOLOGIES INCLUDING IMPACT (IRDIRCS' GOAL 3)*** *(Both paths interconnected* ***continued****)*

- Research on measurement of health outcomes and impact assessment, including utilities and costs.
- Research on quality of life, social needs and tools to support patient-reported outcomes.
- Research on sociological, psychological and economical aspects and impact of RD, assisted and daily life technologies.
- Health & social care services research to improve patient and familial/household health outcomes.
- Development and use of patient reported outcome measures and improvement of outcomes of clinical studies.
- Standardized M-Health based surveillance instruments and of patient entered data (natural history studies, follow-up for treatments and their impact).
- Support new technology solutions & capabilities (i.e. visual recognition technology, smart apps and software that can reduce time and costs).
- Better collection and usage of Real-World Data (RWD) and enhanced methodologies for collection and analysis.
- Information to and training for researchers at all career stages, including clinical/translational research guides and templates, and experiences/best practices' exchange.
- Accelerated translation of research projects' results in clinical studies and healthcare.
- Methodologies for improving research on impact assessment (of different parameters: diagnosis, remaining undiagnosed, treatment, etc).
- Design of broad strategy trials, covering all aspects of patient care besides treatment (care pathway).
- Uncover the potential that computational models and artificial intelligence; big data and block chain.

- Natural history studies and patient registries (also for clinical trial readiness). Related, the improvement of natural history methodologies and economic evaluations, specifically overcoming the deficiencies in research on extremely rare diseases.

- Methodologies for solving cases that are currently difficult to analyse due to different underlying mechanisms (e.g., mosaicism, genomic (non-coding) alterations, gene regulation, complex inheritance), including new genomics / functional genomics technologies, multi-omics, mathematics, biostatistics bioinformatics, and artificial intelligence approaches.

- Measure the (real) impact that the COVID-19 pandemic situation has had (will have) on RD patients at all the levels.

- Measurement of impact of healthcare experiences (e.g., consultations, treatment pathway, coordination, daily life activities) (PREMs).

- Innovative cost-effective methodologies that aid in the scientific evaluation of the efficacy and safety of new therapies and biomarkers.

Box 2: Compilation of mapped needs on Research and Innovation in other related domains (transversal)

Diagnosis & Healthcare	Regulatory & ethics	EU competitiveness & innovation
• Integration & optimization of healthcare pathways, social and everyday needs of RD patients to achieve a holistic care (including access to appropriate treatment, care and psychosocial delivery support in a patient-centered manner).<br>• Overcome barriers related to social justice.<br>• Digital health options, information access, telemedicine, enabling and	• Acceleration of orphan medicines authorisation processes, facilitating the regulatory pathway for potential treatments (including repurposing of drugs) and post-authorisation access.<br>• Identify potential regulatory roadblocks to basket trials.<br>• Facilitate adoption and implementation of RWE in healthcare decision. Marketing	• Facilitate a strategic global collaboration via tools/IA tools/platforms/data-sharing and diagnostic platforms and infrastructures (accessible and coordinated diagnostic ecosystem).<br>• Capacity building to connect innovation, research, and business (training for trainers, to adapt to national schemes, coaching, matchmaking) ; inclusion of early career researchers in calls or networking schemes.

enhancing telehealth for RD across the globe.<br>• Access to diagnostic and medicines.<br>• Advances in personalised medicine.<br>• Effective and equal provision of healthcare for patients with rare diseases.<br>• Capacity building for physicians, patients and families, and knowledge sharing across borders.<br>• High quality care, with new care delivery models, considering sustainability.<br>• Integration of ERNs into Health Systems across Europe.<br>• Reduce the inequality across Europe for people with rare diseases by increasing the availability and accessibility to Orphan products and medical devices.<br>• Reinforcement of private and public sectors' connection on delivery of new technologies.	Authorisation Applications (MAA) based on RWE.<br>• Access to orphan medicines by less developed countries (EU and globally).<br>• Identify barriers to access to standard of care products.<br>• Capacity building on drug development path for pharmaceuticals developers.<br>• Health economics studies through dedicated calls and funding and HTA support, standards and evidence-base.<br>• Identify the scientific, legal and regulatory issues of emerging therapies and technologies, and help with research and development procedures with the support of the EMA and national authorities.<br>• Develop legally and ethically robust agreements for collecting and exchanging health and genetic data, including appropriate consents (ELSI framework) for data sharing and accessible innovative technologies, such as AI.<br>• ELSI research on equality and equity, funding of research, policies, new technologies. Optimisation of data uses regarding its regulatory aspects, harmonising interpretations of the GDPR	• Identify synergies with regional, national, European and international RI and strategies for optimal use.<br>• Increase attractiveness to industry, with clear criteria (financial and non-financial).<br>• Facilitating and improving the data sharing in health research (EC spaces/ ecosystems, e.g. European Health Data Space (TEHDAS), European Health Research and Innovation Cloud (HRIC), OpenScience...).<br>• Optimisation of data and innovative technologies, i.e. artificial intelligence (already scattered in other sections).<br>• The constitution of National Mirror Groups (NMG) (that is also needed out of EU-13 countries).<br>• Identification and attention to the specific EU-13 needs.<br>• Balance between open science and market values in business-like approaches.

	and terms as anonymisation and pseudonymisation by researchers.<br>• Overthrow the heterogeneity and legal basis of national laws/rules on health and research data in addition to GDPR.<br>• Data security and best practices, including collaboration in ethical and legal frame related to this area.<br>• Developing a robust policy framework that encompasses EU and its MS.<br>• Standardisation of data sharing agreements with private sector, which obstructs the public-private collaboration and development of research and innovation.<br>• Adequate balance between private and public sectors and alternative models for funding from private sources.	

Source: Own work, adapted from internal deliverables of the Consortium
