# Supplementary material for: Integrative research and innovation strategy for rare diseases. Insights from the 5-year European joint programme on rare diseases, including analysis to inform recommendations for future actions
Source: Health Res Policy Syst. 2025 Oct 16;23:137. doi: 10.1186/s12961-025-01389-7 (PMC12532414; doi:10.1186/s12961-025-01389-7)
Supplement: Supplementary file 3 — Additional file 3. “Mapped needs covered by EJP RD”: PDF containing the list of mapped needs covered by EJP RD activities [file 12961_2025_1389_MOESM3_ESM.pdf]

Research and Innovation needs covered by EJP RD activities

**DIAGNOSIS PATH IRDIRC GOAL 1**
➢ Easier and more generalized automated exchange of phenotypic data.
➢ Further development of standards and interoperability for data and resources.
➢ Accessible and useful workflows/pipelines for RD data analysis
➢ Collection and linking of high-quality data and biological samples between biobanks and registries (e.g. unique identifiers for patients)
➢ Upgrade of natural history studies, disease progression and mechanisms, including biomarkers.
➢ Identification of clinical biomarkers, clinical outcome measures and surrogate endpoints.
➢ Linking registries and databases to Centers of Expertise.
➢ Improvement and development of diagnostic tests, including better understanding of variants underlying phenotypic difference
➢ Identification of novel pathophysiological pathways in appropriate disease and animal models that effectively mimic the human condition.
➢ Improved annotation and interpretation of variants and development of diagnostic tests for the more prevalent variants.
➢ Novel modalities of functional analysis of candidate variants through in vitro, cell, tissue or animal studies.
➢ Safe deposit of omics data in open or controlled access resources.
➢ Increase of the multi-omics expertise
➢ Omic or multi-omic integrated approaches for discovery of disease causes and mechanisms including development of relevant bioinformatic tools;
➢ New schemes for finding diagnosis for undiagnosed patients, and diagnosis accelerators.
➢ Development and timely adoption of new diagnostic technologies and tools.
➢ Accessible and coordinated diagnostic ecosystem, including healthcare system.
➢ Phenotype-driven diagnosis: integration across different ontologies, integration of shared pathways, digital phenotyping, development of artificial intelligence approaches/applications to extract health related data in aid of diagnosis
➢ Functional strategies to globally stratify variants of unknown significance (VUS) for clinical use; setting up of (in vitro) systems to distinguish between VUS and pathogenic variants (e.g., confirming disruption of splicing for deep intronic variants, loss of protein function, and gain of toxic protein function)
➢ Methodologies for solving cases that are currently difficult to analyse due to different underlying mechanisms (e.g., mosaicism, genomic (non-coding) alterations, gene regulation, complex inheritance), including new genomics / functional genomics technologies, multi-omics, mathematics, biostatistics bioinformatics, and artificial intelligence approaches
**TREATMENT PATH IRDIRC GOAL 2**
➢ Stimulation of novel therapies development through the understanding of the genetic basis, molecular and pathophysiological mechanisms and the natural history of RD.
➢ Optimisation of research on preclinical settings, including proof of concept studies of new therapies, validation of disease models and biomarkers.

➢ Innovative approaches in clinical trial design (adaptive designs, small sizes, clinical end points, repurposing, natural history studies, and precision medicine results, patient-centered, with identification of appropriate biomarkers and surrogate endpoints.
➢ Support to clinical research and registries, at a national and international level through funding, facilities to find patients and patients' registries, biobanks and software.
➢ Framework for basket trials of drugs targeting shared molecular etiologies
➢ Adapt implementation of regulatory requirements, especially for clinical trials in RD.
➢ Earlier research and development of treatments for extremely rare diseases
➢ Creation of guidance and tools on drug repurposing.
➢ Evidence generation for therapies for the very and extremely rare diseases
➢ Acceleration of the research translation into therapies for patients
**PATHS GOAL 1 AND GOAL 2 INTERCONNECTED**
➢ *QUALITY:* Harmonising procedures; harmonised quality requirements for registries and biorepositories; optimisation and innovation in Information & Comm Tech, e-infrastructures, open e-networks, virtual biobanks; establish findable, usable protocols for data collection, infrastructures, data repositories; longitudinal studies, clinical trials and natural history studies, tools and common goals;
➢ *SUSTAINABILITY:* Resources optimization and sharing (multipurpose registries, cluster registries, epidemiological platform and infrastructures); compromises on public funding, e.g. European funding of health networks; sustainable economic models for development and commercialization of orphan drugs
➢ *PATIENT EMPOWERMENT:* Innovative ways for engagement in drug development and clinical trials; patients' needs driven research: better capture of patients' needs; holistic patient-centred approach, inclusion in governance and decision-making setting; capacity building and awareness campaign; options on raising funds for research on their disease and leading their own research projects
➢ *DATA:* Comprehensive and integral health data strategy (e.g. data governance, stewardship) in ERNs & DS platforms; Innovative uses for data, RWD, AI & big data. Improvement of data-linkage from multiple sources and settings; Enhancement of data quality, FAIRness, connectivity, interoperability, with common data sets and ontologies
➢ *NETWORKS, COLLABORATIVE ACTIONS:* Inclusive pan-European multi-stakeholder networks of EU research, patient and healthcare organizations; clinical research networks; collaboration with ERNs on national integration; Open Science (open access to publications, data, and to research data management plans); international framework of collaboration on best practices; IRDIRC global collaboration; Make easier to find suitable facilities or expertise for research (e.g. multi-omics expertise), biobanks/bio samples/cell lines & patients. networks opportunities; enabling of research approaches that combine genetics, environmental and societal challenges; overcome the language barriers, and the lack of a critical mass and role of RD patients (specially EU-13)
**METHODOLOGIES AND IMPACT IRDIRC GOAL 3**
➢ Research on measurement of health outcomes and impact assessment, including utilities and costs.
➢ Research on quality of life, social needs and tools to support patient-reported outcomes.
➢ Research on sociological, psychological and economical aspects and impact of RD, assisted and daily life technologies.
➢ Development and use of patient reported outcome measures and improvement of outcomes of clinical studies.

➢ Standardized M-Health based surveillance instruments and of patient entered data (natural history studies, follow-up for treatments and their impact).
➢ Support new technology solutions & capabilities (i.e. visual recognition technology, smart apps and software that can reduce time and costs).
➢ Information to and training for researchers at all career stages, including clinical/translational research guides and templates, and experiences/best practices' exchange
➢ Methodologies for improving research on impact assessment (of different parameters: diagnosis, remaining undiagnosed, treatment, etc).
➢ Natural history studies and patient registries (also for clinical trial readiness).
➢ Improvement of natural history methodologies and economic evaluations, specifically overcoming the deficiencies in research on extremely rare diseases.
**NON SCIENTIFIC DOMAIN**
**DIAGNOSIS AND HEALTHCARE**
➢ Integration & optimization of healthcare pathways, social and everyday needs of RD patients to achieve a holistic care (including access to appropriate treatment, care and psychosocial delivery support in a patient-centered manner).
➢ Digital health options, information access, telemedicine, enabling and enhancing telehealth for RD across the globe
➢ Access to diagnostic and medicines.
➢ Capacity building for physicians, patients and families, and knowledge sharing across borders.
➢ Integration of ERNs into Health Systems across Europe.
➢ Reinforcement of private and public sectors' connection on delivery of new technologies
**REGULATORY AND ETHICS**
➢ Health economics studies through dedicated calls and funding and HTA support, standards and evidence-base.
➢ Identify the scientific, legal and regulatory issues of emerging therapies and technologies, and help with research and development procedures with the support of the EMA and national authorities.
➢ Develop legally and ethically robust agreements for collecting and exchanging health and genetic data, including appropriate consents (ELSI framework) for data sharing and accessible innovative technologies, such as AI.
➢ ELSI research on equality and equity, funding of research, policies, new technologies
➢ Optimisation of data uses regarding its regulatory aspects, harmonising interpretations of the GDPR and terms as anonymisation and pseudonymisation by researchers (IRDIRC REGUl Y)
➢ Overthrow the heterogeneity and legal basis of national laws/rules on health and research data in addition to GDPR (IRDIRC REGUL Y).
➢ Data security and best practices, including collaboration in ethical and legal frame related to this area
➢ Standardisation of data sharing agreements with private sector, which obstructs the public-private collaboration and development of research and innovation.
➢ Adequate balance between private and public sectors and alternative models for funding from private sources.
**EU COMPETITIVENESS AND INNOVATION**
➢ Facilitate a strategic global collaboration via tools/IA tools/platforms/data-sharing and diagnostic platforms and infrastructures (accessible and coordinated diagnostic ecosystem).

➢ Capacity building to connect innovation, research, and business (training for trainers, to adapt to national schemes, coaching, matchmaking) ; Inclusion of early career researchers in calls or networking schemes.
➢ Identify synergies with regional, national, European and international RI and strategies for optimal use.
➢ Increase attractiveness to industry, with clear criteria (financial and non-financial).
➢ Facilitating and improving the data sharing in health research (EC spaces/ ecosystems, e.g. European Health Data Space (TEHDAS), European Health Research and Innovation Cloud (HRIC), OpenScience...)
➢ The constitution of National Mirror Groups (NMG) (that is also needed out of EU-13 countries)
➢ Identification and attention to the specific EU-13 needs
➢ Balance between open science and market values in business-like approaches

Source: Own work, adapted from internal deliverables of the Consortium
